# Epigenetic silencing of V(D)J recombination is a major determinant for selective differentiation of mucosal-associated invariant t cells from induced pluripotent stem cells

**DOI:** 10.1371/journal.pone.0174699

**Published:** 2017-03-27

**Authors:** Yutaka Saito, Chie Sugimoto, Toutai Mituyama, Hiroshi Wakao

**Affiliations:** 1 Artificial Intelligence Research Center, National Institute of Advanced Industrial Science and Technology (AIST), Koto-ku, Tokyo, Japan; 2 Computational Bio Big-Data Open Innovation Laboratory (CBBD-OIL), National Institute of Advanced Industrial Science and Technology (AIST), Shinjuku-ku, Tokyo, Japan; 3 Core Research for Evolutional Science and Technology (CREST), Japan Science and Technology Agency (JST), Kawaguchi, Saitama, Japan; 4 Department of Hygiene and Cellular Preventive Medicine, Graduate School of Medicine, Hokkaido University, Sapporo, Hokkaido, Japan; Massachusetts General Hospital, UNITED STATES

## Abstract

Mucosal-associated invariant T cells (MAITs) are innate-like T cells that play a pivotal role in the host defense against infectious diseases, and are also implicated in autoimmune diseases, metabolic diseases, and cancer. Recent studies have shown that induced pluripotent stem cells (iPSCs) derived from MAITs selectively redifferentiate into MAITs without altering their antigen specificity. Such a selective differentiation is a prerequisite for the use of MAITs in cell therapy and/or regenerative medicine. However, the molecular mechanisms underlying this phenomenon remain unclear. Here, we performed methylome and transcriptome analyses of MAITs during the course of differentiation from iPSCs. Our multi-omics analyses revealed that recombination-activating genes (*RAG1* and *RAG2*) and DNA nucleotidylexotransferase (*DNTT*) were highly methylated with their expression being repressed throughout differentiation. Since these genes are essential for V(D)J recombination of the T cell receptor (TCR) locus, this indicates that nascent MAITs are kept from further rearrangement that may alter their antigen specificity. Importantly, we found that the repression of *RAG*s was assured in two layers: one by the modulation of transcription factors for *RAG*s, and the other by DNA methylation at the *RAG* loci. Together, our study provides a possible explanation for the unaltered antigen specificity in the selective differentiation of MAITs from iPSCs.

## Introduction

The advent of induced pluripotent stem cells (iPSCs) has enabled the generation of an unlimited number of desired cells upon differentiation for regenerative medicine and/or cell therapy. However, these differentiated cells need to be warranted for proper functionalities and constant identities when clinical applications are envisaged. In the case of T cells, hematopoietic stem cells (HSCs) and embryonic stem cells (ESCs) give rise to immature T cells such as double negative and double positive T cells comprising polyclonal populations harboring a different set of T cell receptors (TCR) [1,2]. TCR are composed of V (D) and J regions that stem from DNA rearrangements of V (D) and J gene segments [3]. V(D)J recombination is mediated by a series of enzymes such as recombination-activating genes 1 and 2 (RAG1 and RAG2) and DNA nucleotidylexotransferase (DNTT). RAG1 and RAG2 recognize signal sequences in V (D) and J segments in genomic DNA, and cleave DNA to rearrange these fragments. DNTT inserts additional nucleotides at the junction (N-region) of the rearranging TCR. Different combinations of V (D) and J gene segments produce TCR with different antigen specificities, thereby enabling T cells to recognize diverse peptidic antigens. However, the polyclonality of T cells has made it difficult to utilize these cells for cell therapy for two reasons. The first issue is intrinsic to the polyclonality of T cells generated from pluripotent cells because the repertoire of TCR is diverse and harbors no specificity to antigens. The second issue is that HSC- and/or ESC-derived T cells still possess the machinery relevant to DNA rearrangements, which may result in further rearrangements in TCR, thereby allowing TCR alternations. In this case, original antigen specificity will be lost, which is inconvenient for cell therapy. Even though the rejuvenation of T cells recognizing specific antigens for HIV and cancer via reprogramming and redifferentiation has been reported, external cues such as anti-CD3/CD28 stimuli have been required to shut down the expression of RAGs and maintain the original TCR [3,4,5]. In contrast, Wakao et al. reported that invariant T cells, called mucosal-associated invariant T cells (MAITs), may be differentiated from iPSCs in a highly selective manner without such external stimuli when iPSCs are prepared from MAITs (MAIT-iPSCs) [6].

MAITs are innate-like T cells harboring an invariant TCRα chain (*TRAV1-2*-*TRAJ33* in both human and mouse), and recognize the vitamin B2 metabolites presented on MHC class I-related protein (MR1) [7]. MAITs play a pivotal role in host defenses against infectious diseases such as bacterial, fungal, and viral infections, and have been implicated in autoimmune and metabolic diseases as well as in cancer, which are often accompanied by the depletion of MAITs from the peripheral blood [7,8,9]. Thus, MAIT cell reprogramming and the selective redifferentiation of MAITs from MAIT-iPSCs are promising strategies for cell therapy and/or regenerative medicine for the above diseases. However, the molecular mechanisms underlying this selective differentiation need to be elucidated in more detail, and proper functionality with an appropriate epigenetic status must be ensured before *in vivo* use.

In the present study, we obtained transcriptome and methylome data relevant to governing T cell identities by comparing the differentiation of T cells from HSC and that of MAITs from MAIT-iPSCs (reMAITs). Our results revealed a difference in the expression of transcripts relevant to the V(D)J recombination machinery concomitant with that in DNA methylation at the corresponding gene loci, which may explain the quasi-exclusive generation of reMAITs from MAIT-iPSCs.

## Results

### Transcriptome and methylome profiling of reMAITs

**Fig 1:**
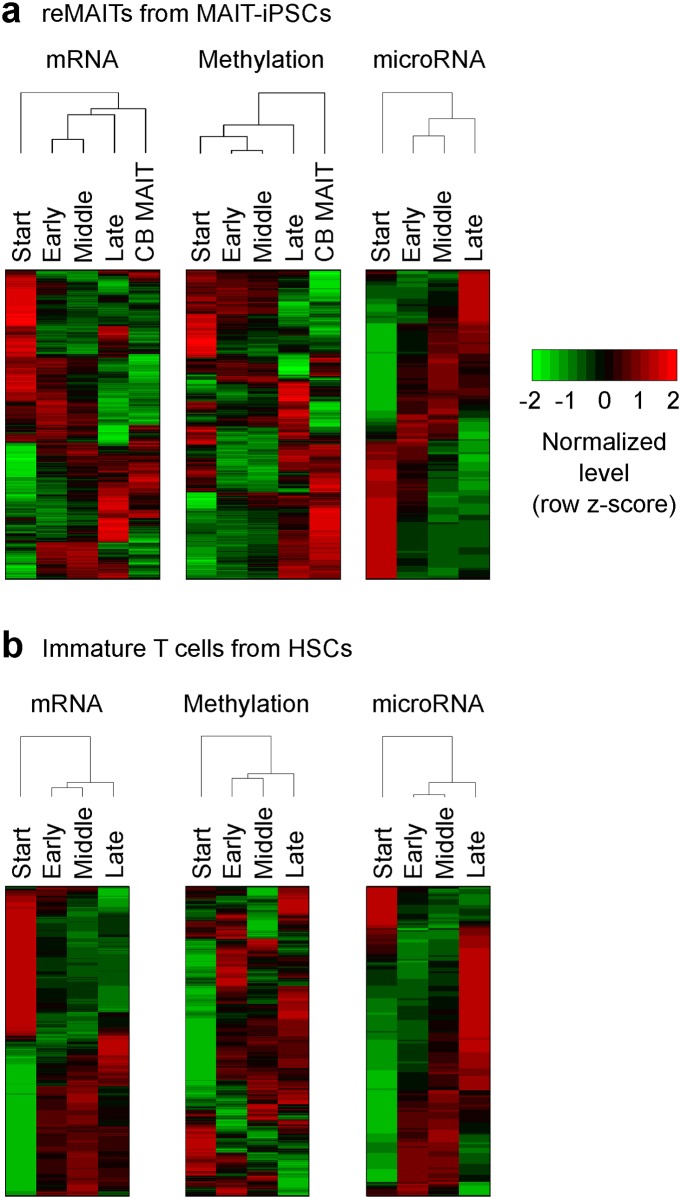
Transcriptome and methylome profiles of reMAITs. (a) reMAITs differentiated from MAIT-iPSCs. Normalized microarray intensities for mRNAs (left), methylation (center), and miRNAs (right). Four time points during the course of reMAIT differentiation are shown (Start, Early, Middle, and Late; See Materials and methods for the definition). Also shown are MAITs isolated from cord blood (CB MAITs). (b) Immature T cells differentiated from HSCs are shown as a control. The top 10% of genes harboring the most time-dependent expression or methylation detected by the limma method are shown. Cluster dendrograms indicate that the transcriptome and methylome change along with differentiation.

In order to elucidate the molecular mechanisms underlying the selective differentiation of reMAITs from MAIT-iPSCs, we conducted transcriptome (mRNAs and microRNAs (miRNAs)) and methylome profiling using microarrays. We sampled reMAITs during the course of differentiation from MAIT-iPSCs as well as MAITs from cord blood (CB MAITs). We used immature T cells differentiated from HSCs as a control. We selected four time points for reMAITs and immature T cells: Start, Early, Middle, and Late (Refer to Materials and methods for a definition of these time points). The transcriptome and methylome of reMAITs both evolved with differentiation, gradually becoming similar to those of CB MAITs (Fig 1a). At the Late stage, the correlation coefficients between reMAITs and CB MAITs were high: 0.909 for gene expression and 0.958 for DNA methylation (S1 Table). These results demonstrated that reMAITs gained similar transcriptome and methylome to CB MAITs as they differentiated [6]. These gradual changes in the transcriptome and methylome were also observed during immature T cell differentiation from HSCs (Fig 1b).

**Fig 2:**
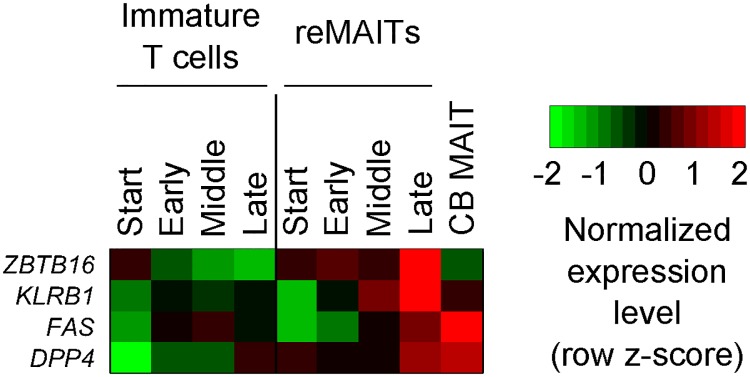
Differential expression of MAIT marker genes. Normalized gene expression levels for the markers of MAITs. Differentially expressed markers (limma, *P* < 0.05) are shown. See S2 Table for the complete list. Marker gene information is obtained from [5].

We then attempted to identify genes that are differentially expressed between reMAITs and immature T cells in order to obtain an insight into the molecular mechanisms governing the T cell fate because the latter consists of polyclonal double negative (CD4^-^CD8^-^) and/or double positive (CD4^+^CD8^+^) T cells, while the former comprises monoclonal cells by the end of differentiation [6]. We found that 4,041 genes were differentially expressed (limma, *P* < 0.05). As expected, these included the hallmarks for MAITs such as *FAS*, *KLRB1* (*CD161*), and *ZBTB16* (*PLZF*) (Fig 2; S2 Table), and were enriched in immunity-related functions such as defense responses (S3 Table).

**Fig 3:**
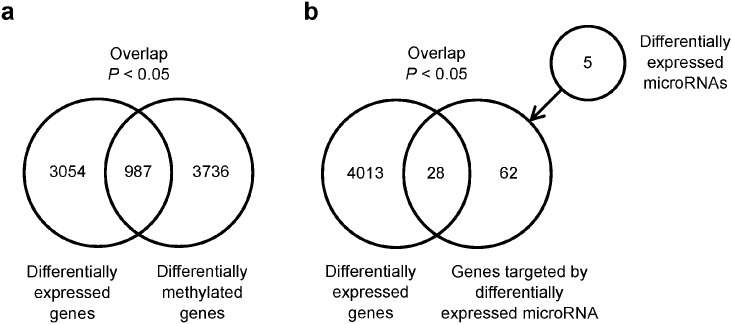
Relationship among differentially expressed genes, differentially methylated genes, and differentially expressed miRNAs between reMAITs and immature T cells. (a) Venn diagram showing differentially expressed genes and differentially methylated genes (limma, *P* < 0.05). (b) Venn diagram showing differentially expressed genes and those targeted by differentially expressed miRNAs (limma, *P* < 0.05). In (a) and (b), the overlap between the two groups is significant (Fisher’s exact test, *P* < 0.05).

Since gene expression is often regulated by DNA methylation, we evaluated the extent to which differential gene expression between reMAITs and immature T cells was attributed to differential DNA methylation (Fig 3a). Specifically, 987 out of 4,041 differentially expressed genes were differentially methylated (limma, *P* < 0.05), showing a correlation between the two groups (Fisher’s exact test, *P* < 0.05). Moreover, differential gene expression was attributed, at least in part, to differential miRNA expression (Fig 3b; S4 Table). Although only five miRNAs were detected as being differentially expressed (limma, *P* < 0.05), miR-146, which is known to function in the innate immune system [10], was detected among them. By using miRNA target information in the miRWalk database [11], we found that the five miRNAs targeted 28 out of 4,041 differentially expressed genes (Fisher’s exact test, *P* < 0.05). These results provide a global view of gene regulation in reMAITs during differentiation for which gene expression was regulated by DNA methylation and miRNAs.

### Suppression of V(D)J recombination in reMAITs

In order to identify the genes responsible for the selective differentiation of reMAITs from MAIT-iPSCs, we focused on genes that showed the differential expression of mRNAs concomitant with differential DNA methylation (Fig 3a). Of note, the genes ranked highest by their statistical significance included *RAG1* and *DNTT* (Table 1), both of which play a pivotal role in V(D)J recombination [3]. In addition, we found that *RAG2*, another gene associated with V(D)J recombination, showed a significant difference in gene expression and DNA methylation (limma, *P* = 0.03 and 0.01, respectively). We also investigated the expression and methylation status of other genes relevant to V(D)J recombination and non-homologous end joining (S5 Table), and confirmed that *RAG*s and *DNTT* were the only genes that showed differential expression concomitant with differential methylation.

**Table 1:** Genes that showed differential expression concomitant with differential DNA methylation between reMAITs and immature T cells.

		*P*-value	
	Description	Differential expression	Differential methylation
CAT	catalase	9.40E-03	1.25E-03
DNTT	deoxynucleotidyltransferase, terminal	9.40E-03	3.93E-02
C22orf34	chromosome 22 open reading frame 34	1.20E-02	2.00E-03
HOXA7	homeobox A7	1.20E-02	4.75E-03
RGS16	regulator of G-protein signaling 16	1.20E-02	4.98E-03
RHAG	Rh-associated glycoprotein	1.20E-02	5.00E-03
D4S234E	DNA segment on chromosome 4 (unique) 234 expressed sequence	1.20E-02	9.46E-03
RAG1	recombination activating gene 1	1.20E-02	9.81E-03
GP9	glycoprotein IX (platelet)	1.20E-02	1.31E-02
MYL4	myosin, light chain 4, alkali; atrial, embryonic	1.20E-02	2.68E-02

Top 10 genes ranked by their statistical significance are shown.

**Fig 4:**
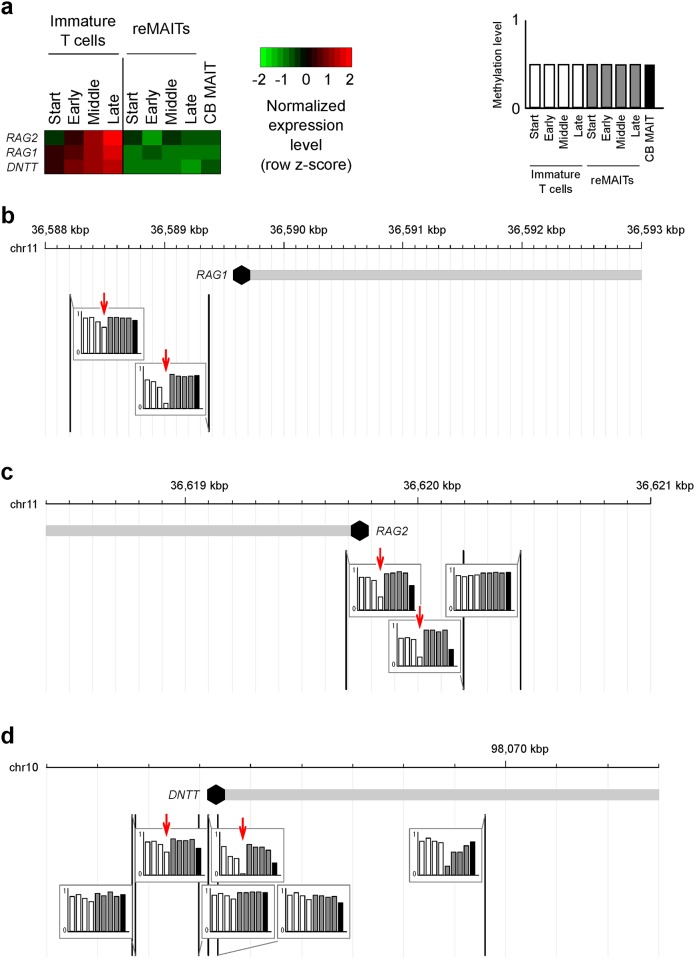
Repression and hypermethylation of genes associated with V(D)J recombination in reMAITs. (a) Normalized gene expression levels for *RAG*s and *DNTT*. (b-d) Methylation status of the *RAG1* (b), *RAG2* (c), and *DNTT* (d) loci. The methylation levels for each cell type are shown in the genome browser snapshots. Vertical lines represent the positions of cytosine residues in the genome. The cytosine residues harboring differential methylation between reMAITs and immature T cells are indicated by red arrows. Transcription start sites are depicted with a closed hexagon in each gene.

The expression levels of *RAG*s and *DNTT* were constantly lower in reMAITs and CB MAITs than in immature T cells throughout differentiation (Fig 4a). Indeed, expression of the mRNA transcripts for *RAG1*, *RAG2*, or *DNTT* in reMAITs was below the detection limit of qPCR (Ct value >45) and at least 450-fold lower than that in immature T cells at any time point (S1 Fig). The experiments were repeated with another preparation of reMAITs and immature T cells, and similar results were obtained. The repression of these genes was accompanied by DNA hypermethylation. The methylation levels of the *RAG1* locus were constantly high in reMAITs, whereas this locus was hypomethylated from the Middle to the Late stages of immature T cell differentiation (Fig 4b). Similar results were obtained at the *RAG2* and *DNTT* loci (Fig 4c and 4d). These results strongly indicate that V(D)J recombination is suppressed in reMAITs, suggesting that the TCR locus in reMAITs is free from further rearrangements that lead to alterations in antigen specificity, namely, T cell identity.

**Fig 5:**
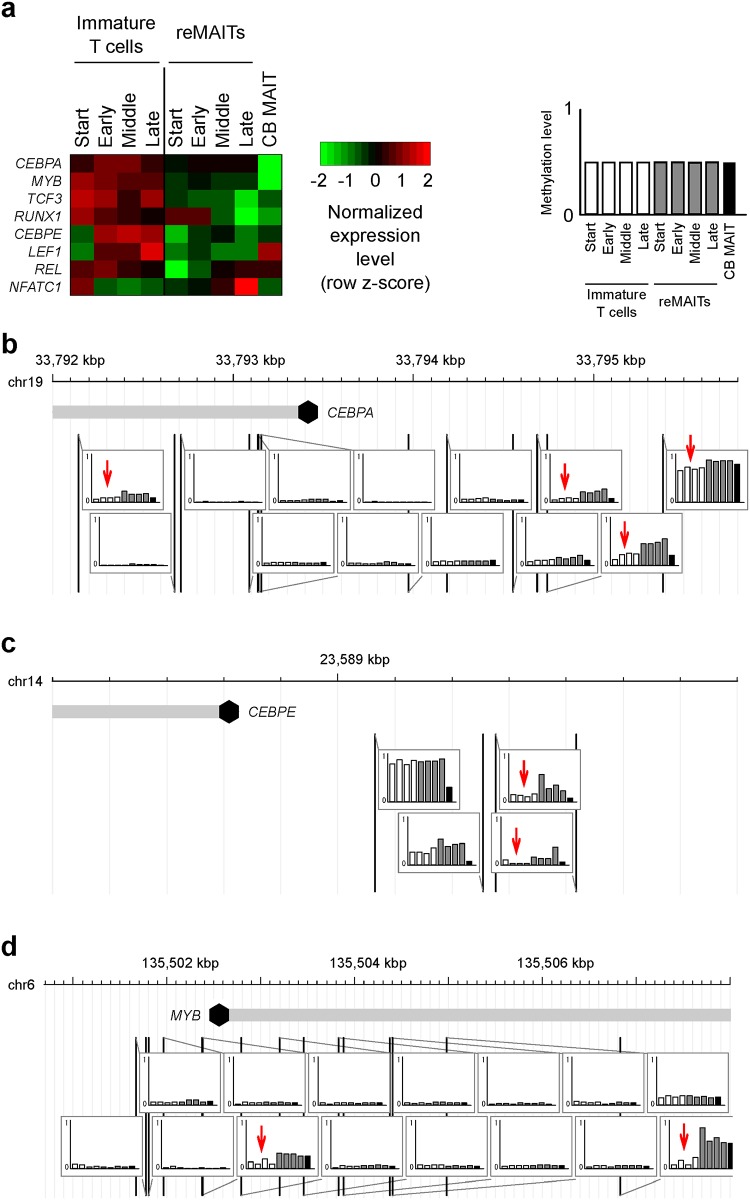
Differential expression of transcription factors known to regulate *RAG*s and their methylation status at the corresponding loci. (a) Normalized gene expression levels for differentially expressed transcription factors (limma, *P* < 0.05). (b-d) Methylation status of the *CEBPA* (b), *CEBPE* (c), and *MYB* (d) loci. The methylation levels for each cell type are shown in the genome browser snapshots. Vertical lines represent the positions of cytosine residues in the genome. The cytosine residues showing differential methylation between reMAITs and immature T cells are indicated by red arrows. Transcription start sites are depicted with a closed hexagon in each gene.

In an attempt to delineate the gene regulatory mechanisms suppressing V(D)J recombination in reMAITs, we also analyzed the expression of transcription factors known to regulate *RAG*s [12]. The expression of transcription factors such as *CEBPA*, *MYB*, *CEBPE*, and *LEF1*, which are known to positively regulate *RAG* expression, was up-regulated in immature T cells (Fig 5a). In contrast, *NFATC1*, which has been shown to repress the expression of *RAG*s [13], was more strongly expressed in reMAITs, in which the expression of *RAG*s was weak. Among these transcription factors, the differential expression of *CEBPA*, *CEBPE*, and *MYB* may be partly attributed to the differential DNA methylation of the corresponding loci. In general, the methylation levels of the *CEBPA* locus were constantly higher in reMAITs than in immature T cells throughout differentiation (Fig 5b). This was also the case for the *CEBPE* and *MYB* loci (Fig 5c and 5d). These results indicate that the repression of *RAG*s in reMAITs was assured not only by DNA methylation at the *RAG* loci, but also by modulating the expression of transcription factors relevant to *RAG* promoter activity.

## Discussion

The results of the present study have provided an insight into the molecular mechanisms underlying the selective differentiation of reMAITs from MAIT-iPSCs. We herein demonstrated that V(D)J recombination was suppressed in reMAITs through the repression of relevant genes such as *RAG*s and *DNTT*. The repression of *RAG*s was achieved at least in two layers: one by transcription factors, and the other by the DNA methylation of loci. This double-layered regulation may ensure that reMAITs are not subjected to further rearrangements that ultimately alter antigen specificity and, thus, T cell identity. This lock mode may be specific to MAITs with semi-invariant TCR because the rejuvenation of HIV and/or cancer antigen-specific T cells via reprogramming accompanies *RAG* [4,5], similar to immature T cells from HSC in this study. Future studies need to clarify what cue(s) or factor(s) trigger differential DNA methylation at *RAGs* and *DNTT* loci, and how they keep these loci hypermethylated during the differentiation of reMAITs from MAIT-iPSCs. The elucidation of such cue(s) or factor(s) may lead to the more efficient differentiation of antigen-specific T cells without further rearrangements in TCR.

Previous studies indicated that TCR rearrangements are regulated not only by *RAG* expression, but also by the epigenetic status of TCR loci [14]. According to this model, the chromatin structure of TCR loci modulates the accessibility of RAG proteins to these loci, thereby regulating rearrangement efficiency. Although this type of regulation has been extensively studied in terms of histone modifications, limited information is currently available on the DNA methylation status of MAITs. Our preliminary data on MAIT-specific TCR loci demonstrated demethylation close to and within the *TRAV1-2* and *TRAJ33* loci, while the loci were heavily methylated in immature T cells (S2 Fig). This difference may be responsible for the strong expression of *TRAV1-2*-*TRAJ33* in reMAITs at the Late stage of differentiation [6]. Further analyses are warranted in order to establish whether these differentially methylated sites affect TCR rearrangement efficiency and influence histone modifications.

Regarding miRNAs, only five miRNAs were found to be differentially expressed between reMAITs and immature T cells (Fig 3b; S4 Table). Such a small number of miRNAs is due, in part, to the relatively limited number of probes available for microarrays (targeting 2,027 miRNAs versus 21,754 mRNAs). Alternatively, miRNAs may have weaker effects on the fate determination of reMAITs than epigenetic regulation such as DNA methylation and transcription factors relevant to *RAG* expression. We were unable to identify any differentially expressed miRNAs that target *RAG*s, *DNTT*, or the transcription factors pertinent to the promoter activity of *RAG*s (limma, *P* < 0.05). Nevertheless, from an omics point of view, the differential gene expression observed between reMAITs and immature T cells may, in part, be attributed to differential miRNA expression (Fig 3b). However, the effects of this differential expression of miRNAs on the destiny of T cells have yet to be elucidated.

Although the transcriptome and methylome of reMAITs at the Late stage became similar to those of CB MAITs from an omics point of view (correlation coefficients of 0.909 for gene expression and 0.958 for DNA methylation; S1 Table), a discrepancy was noted between reMAITs and CB MAITs in terms of the specific genes for MAITs (e.g. *ZBTB16* and *KLRB1* (*CD161*) in Fig 2). This may be due to reMAITs, which differentiated *in vitro*, lacking the appropriate external cue(s) that are a prerequisite for acquiring final differentiation (maturation) concomitant with changes in the transcriptome and epigenome. We previously revealed that the adoptive transfer of reMAITs into mice resulted in migration into different tissues, and this was accompanied by a conversion from the naive to memory type based on cell surface antigens [6]. Thus, in order to realize regenerative medicine and/or cell therapy with reMAITs, the external cue(s) that confer the final maturation of reMAITs with appropriate functionality need to be identified.

In conclusion, this study has provided possible transcriptional and epigenetic mechanisms by which the antigen specificity of MAITs during differentiation from iPSCs was preserved through the suppression of V(D)J recombination. In order to establish whether these mechanisms are applicable to constraining the T cell fate, comprehensive analyses with T cells other than MAITs will be necessary in the future.

## Materials and methods

### Cell culture

Human cord blood (CB) was obtained from the Japanese Red Cross, Hokkaido Block Blood Center with written informed consent. To reduce the influence of individual variability in differential expression/methylation analyses, CB from three different individuals were combined for reMAITs and for immature T cells. reMAITs were prepared as described previously [6]. Briefly, MAIT-iPSCs were cultured on OP9, and CD34^+^CD43^+^ cells were purified with a MACS LS column (Miltenyi Biotech, catalog number 130-042-401). These cells were furthered cultured on OP9/DL1. Based on the reported surface antigen profiles [6], reMAITs were harvested at four different time points after seeding on OP9/DL1: day 0 (Start), day 4 (Early), days 7–10 (Middle), and after day 30 (Late). Regarding the differentiation of immature T cells, CD34^+^ cells from CB were isolated with the CD34 MicroBead Kit (Miltenyi Biotech, catalog number 130-046-702). CD34^+^ HSCs differentiated into the T cell lineage on OP9/DL1, as previously described [15]. Based on surface antigen profiles, immature T cells were harvested at four different time points: CD34^+^ cells at day 0 (Start), day 21 (Early; T lymphocytes remained mostly as CD4^-^CD8^-^ double negative), day 40 (Middle; some lymphocytes (approximately 20–30%) were CD4^+^ CD8^+^ double positive), and after day 50 (Late; most lymphocytes (approximately 70–80%) were CD4^+^CD8^+^ double positive).

### Transcriptome profiling by microarrays

Total RNA was extracted from each sample with an RNeasy MiniKit (Qiagen, catalog number 74104). In the mRNA analysis, RNA was labeled with reverse transcription by incorporating Cy3, and subjected to an analysis using the Human Gene Expression v2 4x44K Microarray Kit (Agilent). In the miRNA analysis, RNA was labeled with Cy3-pCp by ligation, and subjected to an analysis using Human miRNA Microarray Release 19.0 8x60K (Agilent).

### Methylome profiling by Infinium BeadChip

Genomic DNA was extracted from each sample using the Wizard Genomic DNA Purification Kit (Promega, catalog number A1120). DNA (1 μg) was subjected to bisulfite conversion, and subjected to an analysis using the Infinium HumanMethylation450 BeadChip Kit (Illumina).

### qPCR

RNA was isolated using TRIzol reagent (Thermo Fisher Scientific) and cDNA was prepared using SuperScript III First-Strand Synthesis System with random hexamers (Thermo Fisher Scientific). For semi-quantitative real-time PCR, the reaction was performed with FastStart Essential DNA Green Master (Roche) on a LightCycler Nano System (Roche). The PCR primers were described previously [16,17]. The PCR profile was as follows: 95°C for 10 min, 45 cycles of 95°C for 10 sec and 60°C for 30 sec, and the melting curve analysis was performed to verify the amplification specificity. GAPDH was used as housekeeping gene to standardize data, following the ΔCq method.

### Data analysis

Microarray intensities were intra-array and inter-array normalized using limma software [18] for mRNA data, AgiMicroRna software [19] for miRNA data, and IMA software [20] for methylation data. These tools are available as bioconductor packages in the R statistical computing environment. The methylation level for each gene was calculated as the mean methylation level for all probes within -1,500 bp from the transcription start site (denoted by "TSS1500Ind" in the array annotation file). Differential expression and methylation analyses were performed using limma software. For visualization by heatmaps (Figs 1, 2, 4a and 5a), expression levels were log2-transformed, and further transformed into z-scores. Genome browser snapshots (Figs 4b–4d and 5b–5d; S2 Fig) were generated using GenomeTools software [21]. A gene ontology enrichment analysis (S2 Table) was performed using the DAVID web server [22]. The list of genes relevant to V(D)J recombination and non-homologous end joining (S5 Table) was extracted from the GO database ([23]; GO:0033151 and GO:0070419). All *P*-values in statistical tests were corrected for multiple testing by the Benjamini-Hochberg method [24].

## Supporting information

S1 Fig qPCR verification of *RAG*s and *DNTT* expression in immature T cells. qPCR was performed with the primer set specific for *RAG1*, *RAG2*, and *DNTT* as described in the Materials and methods. Relative expression of *RAG1*, *RAG2*, and *DNTT* to that of *GAPDH* at the indicated time is shown. Data are shown with means ± standard deviations (data are measured in triplicate; n = 1). Note that expression levels are shown in a raw value scale, while expression levels in Fig 4a are log2-transformed.
(PDF)

S2 Fig Methylation status in the TCRα locus. The positions of the microarray probes upstream of and within *TRAV1-2* (*Vα7*.*2*) (a) and those downstream of *TRAJ33* (*Jα33*) (b) are shown with the methylation status of the cytosine residue. The position of *TRAJ33* (*Jα33*) is indicated by a red arrow.
(PDF)

S1 Table Relationship between reMAITs and CB MAITs in terms of the transcriptome and methylome. Correlation coefficients between reMAITs and CB MAITs are shown for gene expression and DNA methylation.
(XLSX)

S2 Table Normalized expression levels of MAIT cell marker genes. Differentially expressed genes (limma, *P* < 0.05) between reMAITs and immature T cells are marked by "yes".
(XLSX)

S3 Table Gene Ontology (GO) enrichment analysis of differentially expressed genes between reMAITs and immature T cells. The biological process GO terms are shown with their enrichment *P*-values.
(XLSX)

S4 Table Differentially expressed miRNAs and their target genes. (a) Differentially expressed miRNAs between reMAITs and immature T cells (limma, *P* < 0.05). The target information obtained from the miRWalk database for each miRNA is described. (b) Differentially expressed genes targeted by differentially expressed miRNAs between reMAITs and immature T cells (limma, *P* < 0.05).
(XLSX)

S5 Table Expression and methylation status of genes relevant to V(D)J recombination and non-homologous end joining. For each gene, the statistical significance of differential expression and differential methylation between reMAITs and immature T cells is shown. Note that *RAG*s and *DNTT* were the only genes that showed differential expression concomitant with differential methylation. Not significant: limma, *P* > 0.05.
(XLSX)
