# Supplementary figures and images for: Epigenetic silencing of V(D)J recombination is a major determinant for selective differentiation of mucosal-associated invariant t cells from induced pluripotent stem cells
Source: PLoS One. 2017 Mar 27;12(3):e0174699. doi: 10.1371/journal.pone.0174699 (PMC5367832; doi:10.1371/journal.pone.0174699)

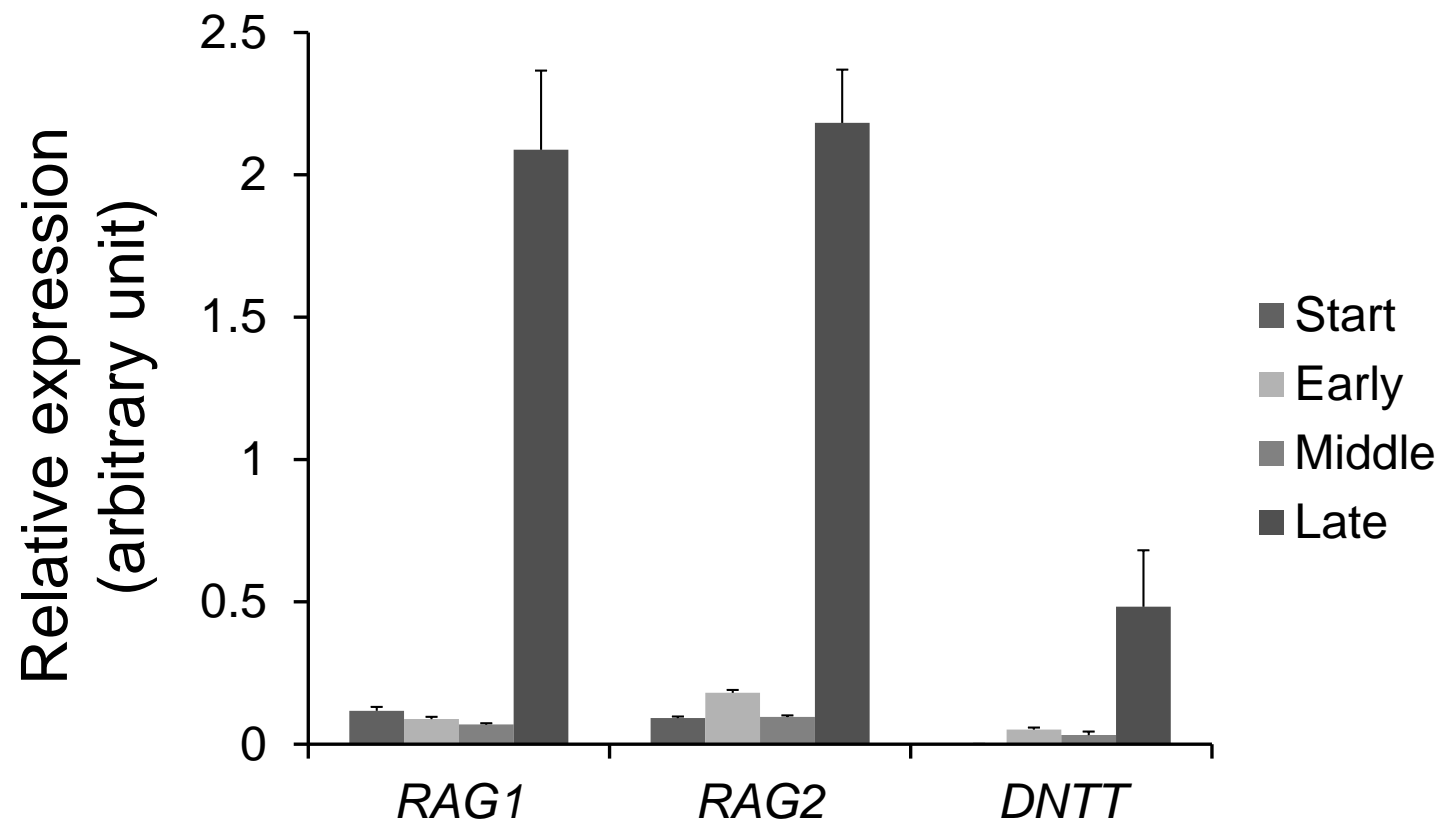

Supplement: S1 Fig — qPCR was performed with the primer set specific for RAG1, RAG2, and DNTT as described in the Materials and methods. Relative expression of RAG1, RAG2, and DNTT to that of GAPDH at the indicated time is shown. Data are shown with means ± standard deviations (data are measured in triplicate; n = 1). Note that expression levels are shown in a raw value scale, while expression levels in Fig 4a are log2-transformed. (PDF) [file pone.0174699.s001.pdf]

**a**

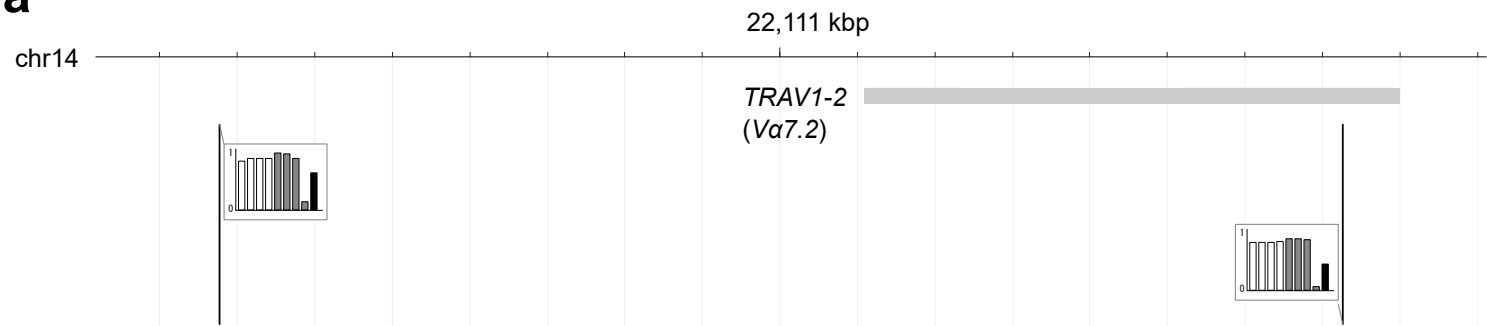

**b**

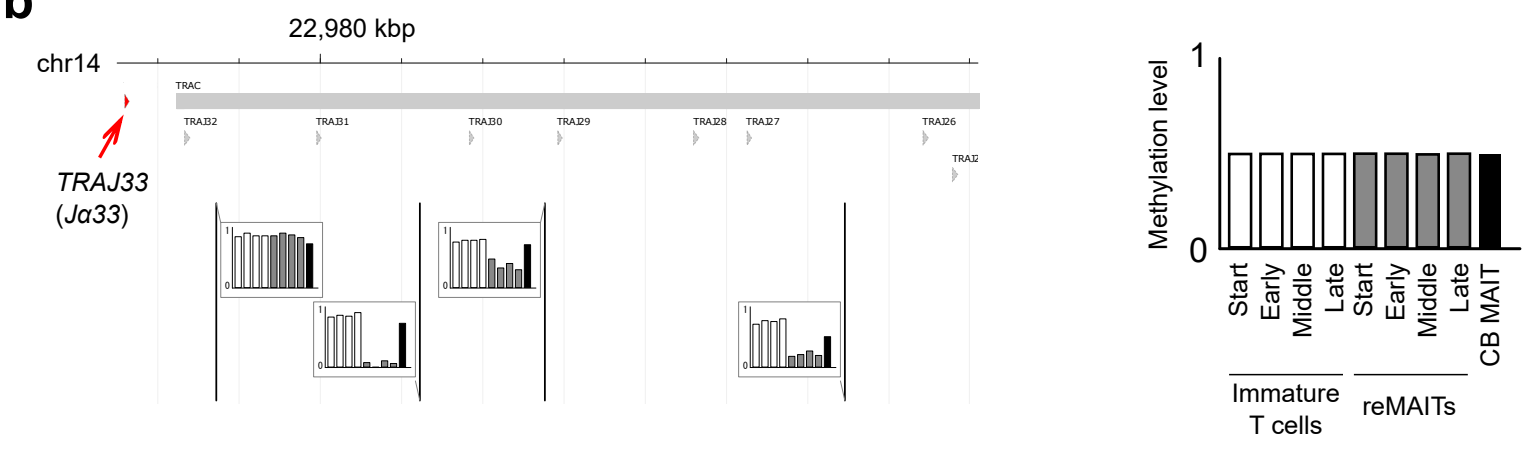

Supplement: S2 Fig — The positions of the microarray probes upstream of and within TRAV1-2 (Vα7.2) (a) and those downstream of TRAJ33 (Jα33) (b) are shown with the methylation status of the cytosine residue. The position of TRAJ33 (Jα33) is indicated by a red arrow. (PDF) [file pone.0174699.s002.pdf]
